# Legume plants may facilitate *Zanthoxylum bungeanum* tolerance to extreme rainfall

**DOI:** 10.1038/s41598-018-34449-w

**Published:** 2018-10-30

**Authors:** Zilong Li, Kaiwen Pan, Akash Tariq, Feng Sun, Sizhong Wang, Lin Zhang, Xiaoming Sun, Xiaogang Wu, Dagang Song

**Affiliations:** 1 CAS Key Laboratory of Mountain Ecological Restoration and Bioresource Utilization & Ecological Restoration Biodiversity Conservation Key Laboratory of Sichuan Province, Chengdu Institute of Biology, Chinese Academy of Sciences, Chengdu, 610041 People’s Republic of China; 2 University of Chinese Academy of Sciences, 100039 Beijing, People’s Republic of China

## Abstract

A complete randomized design was implemented with two watering regimes (extreme rainfall and control) and three different plant combinations (*Zanthoxylum bungeanum*, *Z*. *bungeanum* + *Capsicum annum*, *Z*. *bungeanum* + *Glycine max*) in order to assess the morphological and physio-biochemical responses of focal and neighbor plants. The results indicated that, extreme rainfall had significantly negative impacts on *Z*. *bungeanum* in three intercropping systems. However, intercropping with *G*. *max* improved the transpiration rate (*T*_*r*_) and stomatal conductance (*G*_*s*_), raised leaf relative water content (LRWC), increased chlorophyll a (Chl a) and carotenoid (Car) content, and enhanced the superoxide dismutase activity (SOD) of *Z*. *bungeanum*. After recovery, the *Z*. *bungeanum* + *G*. *max* mixed culture significantly increased soil NO_3_^−^-N, improved the intercellular carbon dioxide concentration (*C*_*i*_) and *T*_*r*_, upregulated soluble sugar and proline, and enhanced hydrogen peroxidase activity (CAT). Moreover, the higher root biomass of *G*. *max* provided much more nitrogen for *Z*. *bungeanum* via the return of organic matter. However, intercropping with *C*. *annum* significantly increased active oxygen (ROS). Compared with neighboring species, in intercropping systems, *G*. *max* could have improved the tolerance of the focal species *Z*. *bungeanum* in response to extreme rainfall and its recovery after extreme rainfall.

## Introduction

Since the late 19^th^ century, the global average surface temperature has increased by 0.85 (0.65–1.06)°C and is predicted to show a continuous warming of >1.5 °C by the end of the century^1^. Global warming is predicted to induce an increase in the frequency and magnitude of extreme rainfall over most regions of the world^2^. Although the mean quantity of precipitation showed no significantly increasing or decreasing trend over 1960–2007 for southwestern China^3^, extreme rainfall events have occured frequently in recent years^4^.

Plants are very vulnerable to extreme precipitation, which has severe impacts on different physiological processes such as photosynthesis water relations or nutrient uptake^5^. Extreme rainfall can decrease photosynthesis, nutrient cycles, and plant productivity^6–8^. Due to excess water, oxygen (O_2_) supply to the root is reduced, and the oxygen demand of plant roots cannot be fulfilled, which reduces root respiration rates, decreases the water absorption and induces stomatal closure^9^. However, the response of individual plants to expected extreme rainfall in relation to neighboring species remains a subject of debate^10^. In a given ecosystem, the patterns in which plants recognize and respond to all aspects of their environment will influence their competitive ability, and thus have important consequences on the overall success of a species.

It is well known that community composition influences plant responses to climate change, with plant-plant interactions playing a key role^11–13^. While they are still not well understood, competition and facilitation are expected to mediate the effects of extreme rainfall^14–16^. Some studies have suggested that, under extreme rainfall, depending on the species, legumes facilitate or compete with the neighboring species^16–18^. Thus, the composition of the companion species is a very important driving factor for the growth and development of a target crop experiencing extreme rainfall. Furthermore, our previous study showed that leguminous plants can stabilize the soil food web via interactions with soil biota communities after extreme rainfall^19^. Unfortunately, there has been limited research on how, in a given ecosystem, neighboring crops impact the stress tolerance of target crops under extreme rainfall^15,20,21^.

As plants provide organic matter to decomposing microbes, the aboveground matter is closely linked with the belowground components. As such, the decomposer subsystem indirectly regulates plant growth and community composition by determining the supply of available soil nutrients^22^. To alleviate the negative effect of climate change, the characteristics of subordinate species are more important for focal species than their plant diversity^23–25^. Therefore, we assumed here that, under extreme rainfall, plant species composition might be crucial in promoting the absorption of nourishment and the physiological processes of focal species.

*Z*. *bungeanum* belongs to the family Rutaceae and is grown widely in India, North America, Australia and southwestern China^17^. The fruits of *Z*. *bungeanum* are medicinal raw materials used for the treatments of toothache and rheumatism and are an important flavoring in Chinese food^26^. It is drought resistant and grows quickly. Due to its important functions, researches into *Z*. *bungeanum* has gained increased attention from scientists^27^.

Most crops grow in soils with low nitrogen, which impacts plant nutrient uptake^28,29^. Tree intercropping systems can enhance environmental and economic benefits, for example, increasing land use efficiency and crop yield^30^ and, conserving biodiversity^31^ and carbon storage^32^. The use of nitrogen-fixing crops can be a strategy to enhance soil fertility^33^. As the third largest crop grown worldwide^34^, soybean is the main source of protein for both humans and animals^35^. Soybeans have the very important feature of being able to establish symbiotic relations with N_2_-fixing soil bacteria^36^. Moreover, soybean has been a well-studied crop with respect to environmental change^37^. For this reason, soybean is the best choice to explain the response of a target plant to environmental change. Hot pepper (*Capsicum annuum* L.) is a vital cash crop and is culinarily and medicinally important. It is also a non-nitrogen fixing plant that is sensitive to moisture^38^. Furthermore, hot pepper requires low organic fertilizer inputs when compared to soybean.

However, *Z*. *bungeanum* is vulnerable to extreme rainfall, which results in declines in yield, quality of fruit and even death^17^. A previous study has indicated that, under extreme rainfall conditions, the leaf nitrogen content of *Z*. *bungeanum* in a mixed legume culture was highest compared to other nonlegume mixed cultures^17^. This is because a significant increase in the root biomass of the soybeans enhanced microbial resistance and the N mineralization rate and promoted the nitrogen absorption of the neighboring crop-*Z*. *bungeanum*^17^. However, there remains a lack of understanding about the physio-biochemical response of *Z*. *bungeanum* to extreme rainfall stress, when grown together in combination with leguminous and nonleguminous plants.

The purpose of this study was to 1) Explore the growth, physiological and biochemical responses of *Z*. *bungeanum* in different intercropping systems under extreme rainfall; 2) Evaluate whether legume species can alleviate the negative impact of extreme rainfall on *Z*. *bungeanum*. To achieve these goals, we studied the plant growth index, photosynthetic traits, pigment content, free radical and antioxidant enzymes in different systems subjected to extreme rainfall. The present study would benefit the management of sustainable agriculture through the development of a reasonable ecosystem with higher resistance to extreme rainfall.

## Result

### Soil properties

After the recovery treatment, under previous normal rainfall conditions, the soil NH_4_^+^-N was higher in the Z-C mixed culture than in the Z monoculture and Z-G mixed culture (*P* < 0.05). Under the previous rainfall treatment, soil NO_3_^−^-N was higher in the Z-G mixed culture than in the Z monoculture and Z-C mixed culture (*P* < 0.05) (Table 1). Planting systems, previous extreme rainfall, and the interactions of the planting system and previous extreme rainfall significantly affected both soil NH_4_^+^-N and NO_3_^−^-N (Table 1).

**Table 1:** Soil physical and chemical properties after recovery. “Z-G” denotes *Z*. *bungeanum* intercropping with *G*. *max*, “Z-C” denotes *Z*. *bungeanum* intercropping with *C*. *annuum*, and “Z” denotes the *Z*. *bungeanum* monoculture.

Treatment stages	Plant system	Z		Z-C		Z-G		System	Rainfall	System*Rainfall
	Traits	Control	Extreme rainfall	Control	Extreme rainfall	Control	Extreme rainfall			
Recovery 30 days	Soil water content (%)	18.03 ± 0.57A	17.82 ± 0.46A	18.24 ± 0.25A	18.79 ± 1.81A	19.24 ± 0.76A	18.33 ± 0.98A	**ns**	**ns**	**ns**
	NH_4_^+^-N (mg kg^−1^)	5.74 ± 0.83B	6.18 ± 1.01AB	10.01 ± 1.07A	9.34 ± 1.43AB	7.28 ± 0.14B	12.45 ± 3.10AB	***	***	***
	NO_3_^−^-N (mg kg^−1^)	3.42 ± 0.24AB	2.37 ± 0.55BC	4.15 ± 0.21A	1.13 ± 0.27C	3.37 ± 0.69A	4.97 ± 0.64A	***	**	***

Different uppercase letters indicate significant differences among all treatments. ANOVA was used to assess the effects of extreme rainfall on soil properties. *P < 0.05, **P < 0.01, ***P < 0.001, ^ns^non-significant (P > 0.05).

### Nitrogen content of plant leaves

Irrespective of extreme rainfall, when compared with the Z-G mixed culture, the NH_4_^+^-N content of the *Z*. *bungeanum* leaves decreased significantly in the Z monoculture and Z-C mixed culture. Extreme rainfall significantly decreased the NO_3_^−^-N content of *G*. *max*. The two-way ANOVA showed that planting systems, extreme rainfall, and their interaction significantly affected the NH_4_^+^-N content of the *Z*. *bungeanum* leaves (Table 2).

**Table 2:** Leaf nitrogen content of *Z*. *bungeanum* and neighbor crops. “Z-G” denotes *Z*. *bungeanum* intercropping with *G*. *max*, “Z-C” denotes *Z*. *bungeanum* intercropping with *C*. *annuum*, and “Z” denotes the *Z*. *bungeanum* monoculture. Different uppercase letters indicate significant differences among all treatments.

Plant system	Z		Z-C		Z-G		System	Rainfall	System* Rainfall
Traits	Control	Extreme rainfall	Control	Extreme rainfall	Control	Extreme rainfall			
NH_4_^+^-N content of *Z*. *bungeanum* leaves (mg g^−1^)	5.61 ± 0.68B	3.24 ± 0.17C	4.60 ± 0.49B	3.97 ± 0.25C	8.71 ± 0.38A	5.44 ± 0.29B	***	***	*
NH_4_^+^-N content of neighbor crops leaves (mg g^−1^)			2.60 ± 0.11A	2.46 ± 0.20A	2.65 ± 0.33A	2.44 ± 0.14A	**/**	**/**	**/**
NO_3_^−^-N content of *Z*. *bungeanum* leaves (mg g^−1^)	13.03 ± 0.62A	12.16 ± 0.67A	12.78 ± 0.73A	10.81 ± 0.98A	13.08 ± 1.05A	13.85 ± 1.01A	ns	ns	ns
NO_3_^−^-N content of neighbor crops leaves (mg g^−1^)			20.14 ± 1.37A	14.89 ± 0.96A	17.36 ± 1.33A	9.93 ± 0.83B	**/**	**/**	**/**

ANOVA was used to assess the effects of extreme rainfall on leaf nitrogen content. *P < 0.05, **P < 0.01, ***P < 0.001, ^ns^non-significant (P > 0.05).

### Focal plant growth and neighbor species biomass

Under normal water conditions, the LRWC of *Z*. *bungeanum* decreased significantly in the Z-C culture when compared with the monoculture and Z-G mixed culture (*P* < 0.05) (Table 3). Extreme rainfall reduced LRWC and the height of *Z*. *bungeanum* in all cultures, and it significantly decreased the aboveground weight of *G*. *max and C*. *annum* (*P* < 0.01). A two-way ANOVA showed that the planting system significantly affected the LRWC of *Z*. *bungeanum* (*P* < 0.001) and that the extreme rainfall significantly affected the LRWC and height of *Z*. *bungeanum* (*P* < 0.05) (Table 3).

**Table 3:** Effects on the growth parameters of *Z*. *bungeanum* and neighbor crops after treatment and recovery. “Z-G” denotes *Z*. *bungeanum* intercropping with *G*. *max*, “Z-C” denotes *Z*. *bungeanum* intercropping with *C*. *annuum*, and “Z” denotes the *Z*. *bungeanum* monoculture. “LRWC” denotes leaf relative water content.

Treatment stages	Plant system	Z		Z-C		Z-G		System	Rainfall	System* Rainfall
	Traits	Control	Extreme rainfall	Control	Extreme rainfall	Control	Extreme rainfall			
30 day	LRWC (100%)	0.86 ± 0.02A	0.81 ± 0.02AB	0.70 ± 0.08B	0.65 ± 0.02B	0.86 ± 0.02A	0.79 ± 0.08AB	***	*	ns
	Height (cm)	151.0 ± 11.15A	142.7 ± 8.84AB	180.0 ± 15.31A	111.5 ± 6.64B	158.0 ± 27.51A	126.0 ± 14.00AB	ns	*	ns
	Aboveground weight of neighbor crops (g)			6.26 ± 0.33B	3.53 ± 0.24C	9.68 ± 0.51A	3.57 ± 0.32C	/	/	/
Recovery 30 day	LRWC (100%)	0.53 ± 0.02B	0.58 ± 0.01B	0.53 ± 0.00B	0.59 ± 0.05B	0.67 ± 0.06A	0.64 ± 0.11AB	ns	ns	ns
	Height (cm)	165.0 ± 7.57A	152.7 ± 2.73A	175.0 ± 13.86A	123.0 ± 13.31A	155.3 ± 28.18A	147.7 ± 9.26A	ns	ns	ns
	Yield (g)			90.83 ± 0.38A	24.47 ± ± 4.50B	3.89 ± 1.21C	1.00 ± 0.31D	/	/	/
	Root biomass of neighbor crops (g)			8.70 ± ± 2.14A	7.32 ± 1.54A	4.46 ± 0.52B	8.20 ± 0.89A	/	/	/
	Stem and leaf weight of neighbor crops (g)			7.66 ± 0.27B	7.44 ± 0.56B	22.38 ± 1.87A	8.03 ± 1.08B	/	/	/

Different uppercase letters indicate significant differences among all treatments. ANOVA was used to assess the effects of extreme rainfall on plant growth. *P < 0.05, **P < 0.01, ***P < 0.001, ^ns^non-significant (P > 0.05).

After recovery treatments, the LRWC of *Z*. *bungeanum* in the Z-G mixed culture was higher than that in the Z-C mixed culture and Z monoculture (*P* < 0.05). The root biomass of *G*. *max* previously treated with extreme rainfall was significantly larger than that of the control after recovery (*P* < 0.05). The previous extreme rainfall significantly reduced the yield of *C*. *annuum* and *G*. *max* (*P* < 0.01). A two-way ANOVA showed that after one month of the recovery treatment, the planting system did not have significant effects on the growth of *Z*. *bungeanum* (Table 3).

### Photosynthetic parameters

Under the normal rainfall treatment, the *C*_i_ of *Z*. *bungeanum* in Z-C mixed culture decreased significantly in comparison with the Z-G mixed culture and Z monoculture. The extreme rainfall treatment significantly reduced the *P*_n_ of *Z*. *bungeanum* in the Z monoculture, Z-C and Z-G mixed cultures by 12.83%, 25.71% and 22.96%, respectively (*P* < 0.05). The *T*_*r*_ of *Z*. *bungeanum* in the Z-C mixed culture was significantly smaller than that in the Z-G mixed culture and Z monoculture under extreme rainfall (*P* < 0.05). The extreme rainfall treatment significantly decreased the *T*_*r*_ of *Z*. *bungeanum* in the Z-C mixed culture by 30.27% (*P* < 0.05). The planting systems significantly affected the *P*_*n*_, *G*_*s*_, *C*_*i*_ and *T*_*r*_; the extreme rainfall significantly affected the *P*_*n*_ and *T*_*r*_ (*P* < 0.001). There was a significant interaction effect between the planting system and extreme rainfall on *T*_*r*_ (Table 4). The order of the effects on the photosynthetic parameters of *Z*. *bungeanum* is planting system > extreme rainfall > interaction of planting system and extreme rainfall.

**Table 4:** Effects on the photosynthetic parameters of *Z*. *bungeanum* after treatment and recovery. “Z-G” denotes *Z*. *bungeanum* intercropping with *G*. *max*, “Z-C” denotes *Z*. *bungeanum* intercropping with *C*. *annuum*, and “Z” denotes *Z*. *bungeanum* monoculture. “*P*_*n*_” denotes net photosynthetic, “*G*_*s*_” denotes stomatal conductance, “*C*_*i*_” denotes intercellular carbon dioxide concentration, “*T*_*r*_” denotes transpiration rate.

Treatment stages	Plant system	Z		Z-C		Z-G		System	Rainfall	System*Rainfall
	Traits	Control	Extreme rainfall	Control	Extreme rainfall	Control	Extreme rainfall			
30 days	*Pn* (μmol m^−2^ s^−1^)	15.36 ± 0.03A	13.39 ± 0.16B	14.39 ± 1.15A	10.69 ± 0.59C	13.94 ± 0.87A	10.74 ± 0.73C	**	***	ns
	*Gs* (mmol m^−2^ s^−1^)	0.32 ± 0.01A	0.29 ± 0.02A	0.19 ± 0.04A	0.18 ± 0.03AB	0.32 ± 0.06A	0.31 ± 0.00A	**	ns	ns
	*Ci* (μmol mol^−1^)	305.06 ± 5.47A	279.54 ± 12.69A	271.15 ± ± 3.57AB	269.35 ± 0.38AB	302.40 ± 10.86A	294.72 ± 5.52A	**	ns	ns
	*Tr* (g m^−2^ h^−1^)	3.55 ± 0.18B	3.56 ± 0.01B	3.70 ± 0.03B	2.58 ± 0.14C	3.56 ± 0.15B	4.05 ± 0.04A	*	***	***
Recovery 30 days	*Pn* (μmol m^−2^ s^−1^)	7.45 ± 0.93B	7.78 ± 0.59B	13.57 ± 0.38A	7.91 ± 2.34B	10.96 ± 1.28A	9.24 ± 1.18AB	**	*	*
	*Gs* (mmol m^−2^ s^−1^)	0.13 ± 0.02A	0.13 ± 0.00A	0.11 ± 0.03A	0.13 ± 0.04A	0.13 ± 0.01A	0.22 ± 0.41A	ns	*	ns
	*Ci* (μmol mol^−1^)	261.48 ± 16.7A	249.47 ± 21.6AB	279.04 ± 30.3AB	223.08 ± 34.9BC	311.90 ± ± 13.67A	310.40 ± 0.6A	*	ns	ns
	*Tr* (g m^−2^ h^−1^)	1.31 ± 0.10A	1.23 ± 0.05A	0.80 ± 0.08C	1.06 ± 0.01B	0.77 ± 0.12C	1.13 ± 0.01B	***	**	*

Different uppercase letters indicate significant differences between the control (normal rainfall) treatments; different lowercase letters indicate significant differences between the extreme rainfall treatments. ANOVA was used to assess the effects of extreme rainfall on photosynthetic parameters. *P < 0.05, **P < 0.01, ***P < 0.001, ^ns^non-significant (P > 0.05).

After recovery with the previous normal rainfall treatment, the *P*_*n*_ of *Z*. *bungeanum* of the Z-C and Z-G mixed cultures increased significantly (*P* < 0.05), while the *T*_*r*_ of *Z*. *bungeanum* in the Z-C and Z-G mixed cultures decreased significantly in comparison with the Z monoculture (*P* < 0.05). After recovery with the previously extreme rainfall, the *C*_i_ of the *Z*. *bungeanum* in the Z-C mixed culture was significantly lower than that in the Z-G mixed culture (*P* < 0.05). Irrespective of whether the previous treatments were of normal or extreme rainfall, the *T*_*r*_ of *Z*. *bungeanum* in the Z monoculture was significantly higher than that in the Z-C and Z-G mixed cultures (*P* < 0.05). The planting system significantly affected the *P*_*n*_, *C*_*i*_ and *T*_*r*_; the extreme rainfall significantly affected the *P*_*n*_, *G*_*s*_ and *T*_*r*_. There was a significant interaction effect between the planting system and extreme rainfall on *P*_*n*_ and *T*_*r*_ (Table 4). Either at the stage of extreme rainfall or at the recovery phase, the *T*_*r*_ was the most affected parameter.

### Photosynthetic pigments

Under normal rainfall conditions for all cultures, no significant difference was found in the Car content of the *Z*. *bungeanum* leaves. Irrespective of normal or extreme rainfall treatments, the Chl a/Chl b of the *Z*. *bungeanum* leaves of the Z-C mixed culture and Z monoculture was significantly lower when compared with Z-G (*P* < 0.05). Under extreme rainfall, in comparison with the Z monoculture, the content of Chl a, Chl b and Car, and Chl a/Chl b of *Z*. *bungeanum* in the Z-G mixed culture significantly increased by 52.74%, 24.30%, 31.35% and 23.47%, respectively (*P* < 0.05). There were no significant differences in the Chl a, Chl b and Car contents, and the Chl a/Chl b of *Z*. *bungeanum* between the Z-C mixed culture and Z monoculture. The planting system significantly affected the Chl a and Car contents and the Chl a/Chl b; the extreme rainfall significantly affected the content of Car, while the planting system and extreme rainfall interactively impacted the content of Chl a and Chl b (*P* < 0.01). In the extreme rainfall phase, the influence of extreme rainfall and that of the interaction of the planting system and extreme rainfall were more obvious that of the planting system alone (Table 5).

**Table 5:** Effects on pigment parameters of *Z*. *bungeanum* after treatment and recovery. “Z-G” denotes *Z*. *bungeanum* intercropping with *G*. *max*, “Z-C” denotes *Z*. *bungeanum* intercropping with *C*. *annuum*, and “Z” denotes the *Z*. *bungeanum* monoculture. “Chl a” denotes chlorophyll a, “Chl b” denotes chlorophyll b, and “Car” denotes carotenoid. Different uppercase letters indicate significant differences among all treatments.

Treatment stages	Plant system	Z		Z-C		Z-G		System	Rainfall	System* Rainfall
	Traits	Control	Extreme rainfall	Control	Extreme rainfall	Control	Extreme rainfall			
30 days	Chl a (mg g^−1^ FW)	4.86 ± 0.47B	4.57 ± 0.09B	4.69 ± 0.10B	4.47 ± 0.02B	4.97 ± 0.36B	6.98 ± 0.37A	**	ns	**
	Chl b (mg g^−1^ FW)	2.25 ± 0.06A	2.14 ± 0.06AB	2.86 ± 0.22A	2.37 ± 0.16A	1.70 ± 0.30B	2.66 ± 0.12A	ns	ns	**
	Car (mg g^−1^ FW)	1.54 ± 0.23B	1.85 ± 0.06B	2.07 ± 0.17B	1.94 ± 0.11B	1.90 ± 0.04B	2.43 ± 0.07A	*	*	ns
	Chl a/Chl b	2.16 ± 0.16B	2.13 ± 0.07B	1.66 ± 0.16B	1.91 ± 0.13B	3.04 ± 0.35A	2.63 ± 0.02A	***	ns	ns
Recovery 30 days	Chl a (mg g^−1^ FW)	4.27 ± 0.24B	5.33 ± 0.19A	3.44 ± 0.13C	4.74 ± 0.07A	3.97 ± 0.16BC	4.68 ± 0.72AB	ns	**	ns
	Chl b (mg g^−1^ FW)	2.02 ± 0.11C	2.44 ± 0.07AB	1.54 ± 0.11D	3.24 ± 0.34A	2.10 ± 0.03BC	2.31 ± 0.25B	ns	***	**
	Car (mg g^−1^ FW)	1.83 ± 0.03B	2.23 ± 0.09A	1.51 ± 0.03C	1.83 ± 0.16BC	2.23 ± 0.11A	2.16 ± 0.06AB	***	*	ns
	Chl a/Chl b	2.11 ± 0.01A	2.18 ± 0.01A	2.24 ± 0.08A	1.49 ± 0.15C	1.89 ± 0.10B	2.02 ± 0.19AB	ns	ns	**

ANOVA was used to assess the effects of extreme rainfall on leaf pigment content. *P < 0.05, **P < 0.01, ***P < 0.001, ^ns^non-significant (*P* > 0.05).

After recovery, the previously extreme rainfall treatment significantly increased the contents of Chl a, Chl b and Car of the *Z*. *bungeanum* leaves in the Z monoculture (*P* < 0.05). There were no significant differences in the Chl a, Chl b, and Car content and Chl a/Chl b of *Z*. *bungeanum* in the Z-G between the previously extreme rainfall treatment and control. The Chl a and Chl b contents of *Z*. *bungeanum* in the Z-C mixed culture with the previously extreme rainfall treatment increased significantly (*P* < 0.01), while the Chl a/Chl b significantly decreased. The planting systems significantly affected the Car content (*P* < 0.001), and the previously extreme rainfall significantly affected the Chl a, Chl b and Car contents, while the interaction of the planting system and extreme rainfall significantly affected the Chl b content and Chl a/Chl b (*P* < 0.01).

### ROS production and lipid peroxidation

**Figure 1:**
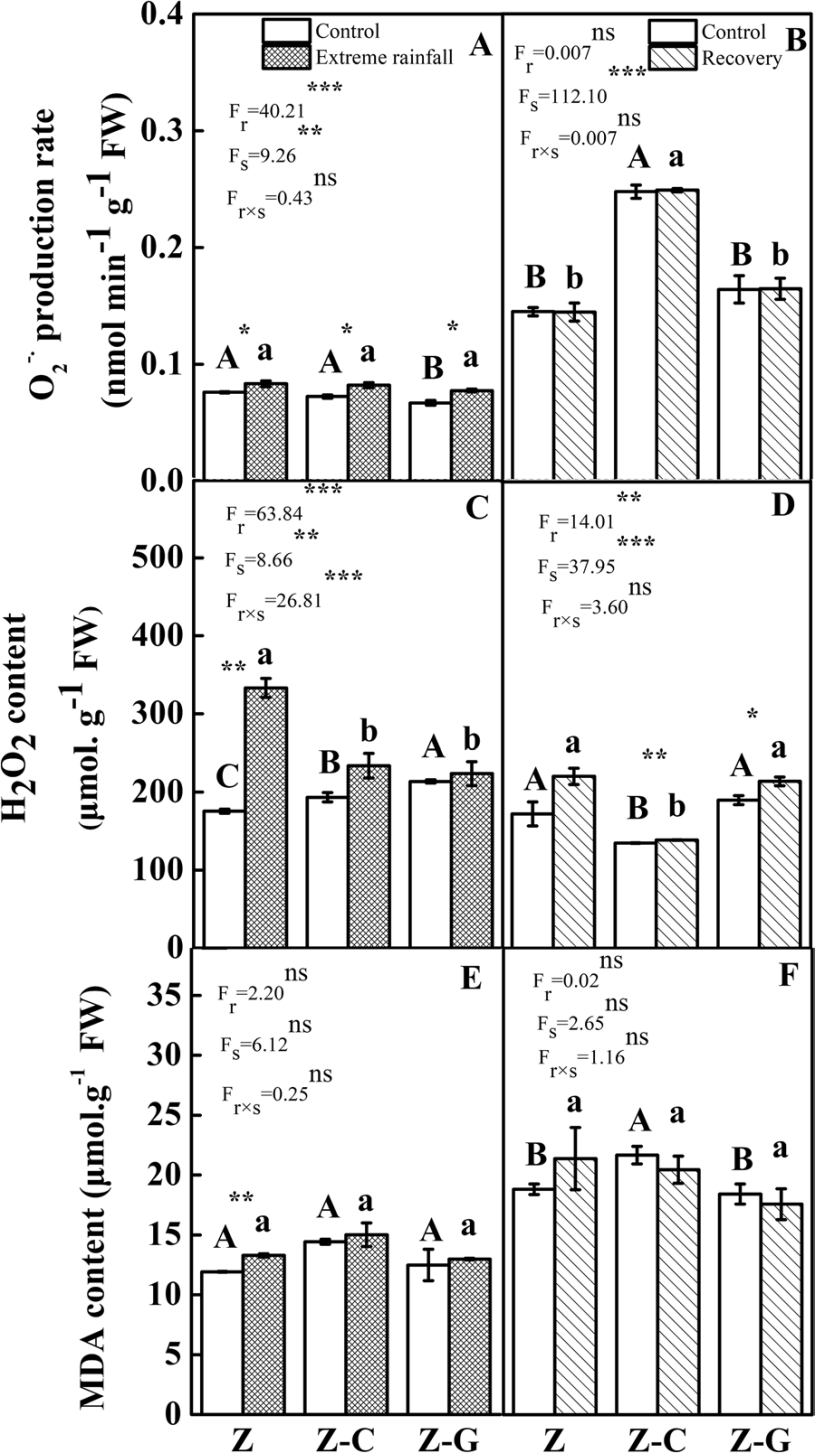
Effects of free radical and MDA content on focal species after extreme rainfall and recovery. The left column represents 30 days of rainfall, the right column represents recovery for 30 days; “Z-G” denotes *Z*. *bungeanum* intercropping with *G*. *max*, “Z-C” denotes *Z*. *bungeanum* intercropping with *C*. *annuum*, and “Z” denotes the *Z*. *bungeanum* monoculture. Vertical bars show ± SE of the mean (n± SE). Different uppercase letters indicate significant differences between the control (normal rainfall) treatments; different lowercase letters indicate significant differences between the extreme rainfall treatments. “r” denotes extreme rainfall; “s” denotes planting system; “r × s” denotes the interaction of extreme rainfall and planting system; Proportion of explained variance by extreme rainfall and planting system effects and by their interactions (two-way ANOVA). Significant levels: *P < 0.05, **P < 0.01, ***P < 0.001, “ns” no significant.

Under normal rainfall, the O_2_^−·^. production rate of the *Z*. *bungeanum* leaves of the Z-G mixed culture decreased significantly (*P* < 0.05), while the H_2_O_2_ content increased significantly compared with the Z-C mixed culture and Z monoculture (*P* < 0.05) (Fig. 1A,C). The extreme rainfall significantly increased the O_2_^−·^. production rate of the *Z*. *bungeanum* leaves by 9.21%, 12.33% and 16.42% (*P* < 0.05); raised the H_2_O_2_ content by 89.69%, 20.94% and 4.73%; and increased the malondialdehyde (MDA) content by 11.49%, 4.09% and 4.00% in the Z monoculture, Z-C and Z-G mixed cultures, respectively. The two-way ANOVA showed that the planting system and extreme rainfall both significantly affected the O_2_^−·^. production rate and H_2_O_2_ content. The interaction effects of planting systems and extreme rainfall significantly affected H_2_O_2_ content (*P* < 0.001) (Fig. 1A,C).

After recovery with the previously normal rainfall treatment, the O_2_^−·^. production rate and MDA content of the *Z*. *bungeanum* leaves of the Z-C mixed culture increased significantly while the H_2_O_2_ content decreased significantly compared with the Z monoculture and Z-G mixed cultures (*P* < 0.05) (Fig. 1B,D,F). However, after recovery with the previously extreme rainfall treatment, the O_2_^−·^. production rate of *Z*. *bungeanum* increased significantly while, compared with the Z monoculture and Z-G mixed cultures, the H_2_O_2_ content decreased significantly in the Z-C mixed culture (*P* < 0.05). The planting system significantly affected the O_2_^−·^. production rate and H_2_O_2_ content (*P* < 0.001) (Fig. 1B–D).

### Antioxidant stress components

**Figure 2:**
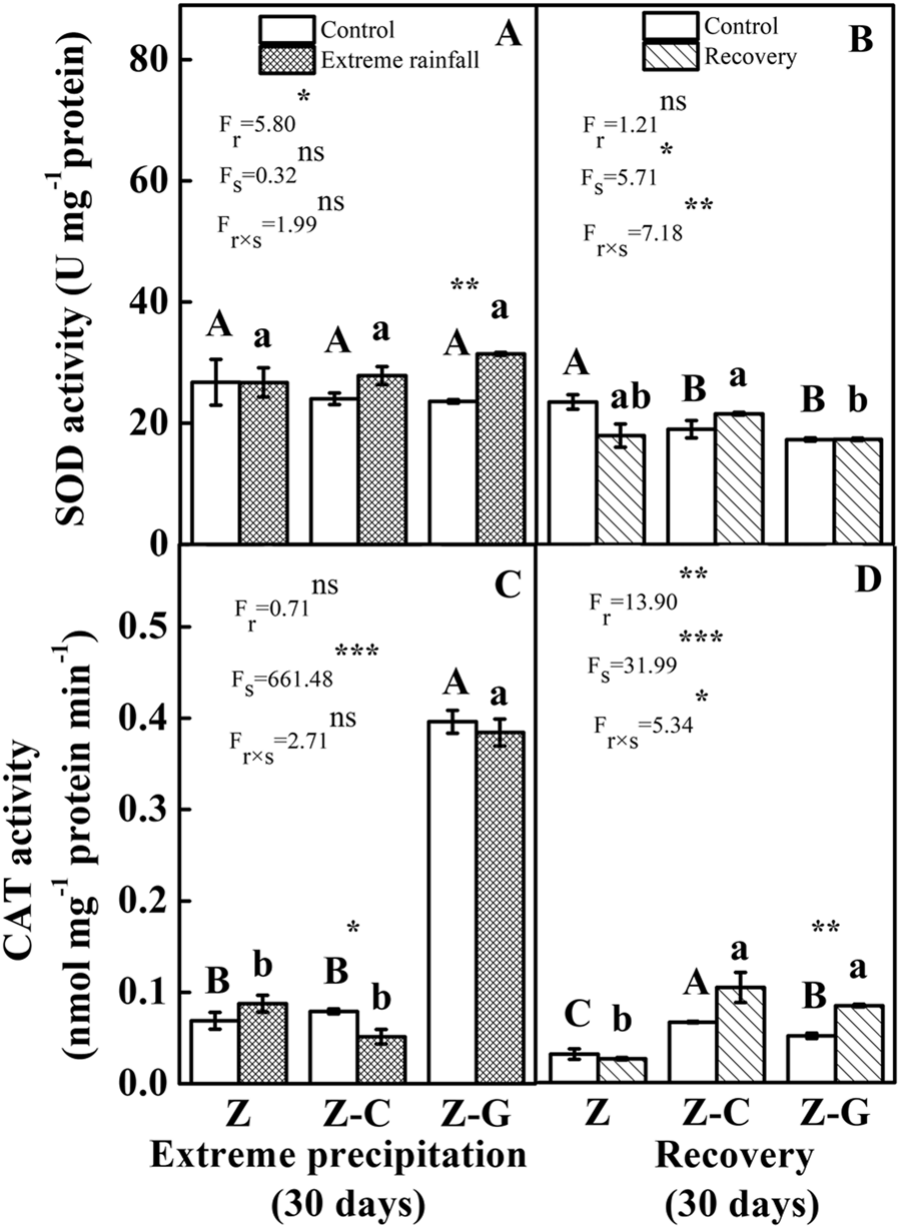
Effects of leaf SOD and CAT activity on focal species after extreme rainfall and recovery. The left column represents 30 days of rainfall, the right column represents recovery for 30 days; “Z-G” denotes *Z*. *bungeanum* intercropping with *G*. *max*, “Z-C” denotes *Z*. *bungeanum* intercropping with *C*. *annuum*, and “Z” denotes the *Z*. *bungeanum* monoculture. Vertical bars show ± SE of the mean (n± SE). Different uppercase letters indicate significant differences between the control (normal rainfall) treatments; different lowercase letters indicate significant differences between the extreme rainfall treatments. “r” denotes extreme rainfall; “s” denotes planting system; “r × s” denotes the interaction of extreme rainfall and planting system; Proportion of explained variance by extreme rainfall and planting system effects and by their interactions (two-way ANOVA). Significant levels: *P < 0.05, **P < 0.01, ***P < 0.001, “ns” no significant.

Irrespective of the normal or extreme rainfall treatments, no significant difference of SOD activity in *Z*. *bungeanum* leaves was found between the Z monoculture, Z-C and Z-G mixed cultures (Fig. 2A). The extreme rainfall treatment significantly raised the SOD activity of the *Z*. *bungeanum* leaves in the Z-G mixed culture by 33.21% (*P* < 0.05). Compared to the Z monoculture, and Z-C under normal rainfall and extreme rainfall treatments, the CAT activity of the *Z*. *bungeanum* leaves increased significantly in the Z-G mixed culture (Fig. 2C). Extreme rainfall significantly affected the SOD activity of the *Z*. *bungeanum* leaves (*P* < 0.05), the planting system significantly affected the CAT activity of the *Z*. *bungeanum* leaves (*P* < 0.001) (Fig. 2A–C).

After recovery under the previously extreme rainfall treatment, the SOD activity of the *Z*. *bungeanum* leaves of Z-C was significantly higher than that of Z-G (Fig. 2B). Under the previously normal rainfall condition, the Z monoculture had higher SOD activity compared to the Z-C and Z-G mixed cultures. Irrespective of previously normal or extreme rainfall treatments, the Z-C and Z-G mixed cultures had higher CAT activity than the Z monoculture (Fig. 2D). In the Z-G mixed culture, the previously extreme rainfall treatment significantly increased the CAT activity by 64.71%. The interaction effects of planting systems and extreme rainfall significantly affected the SOD and CAT activity of the *Z*. *bungeanum* leaves (Fig. B,D).

### Biochemical parameters

**Figure 3:**
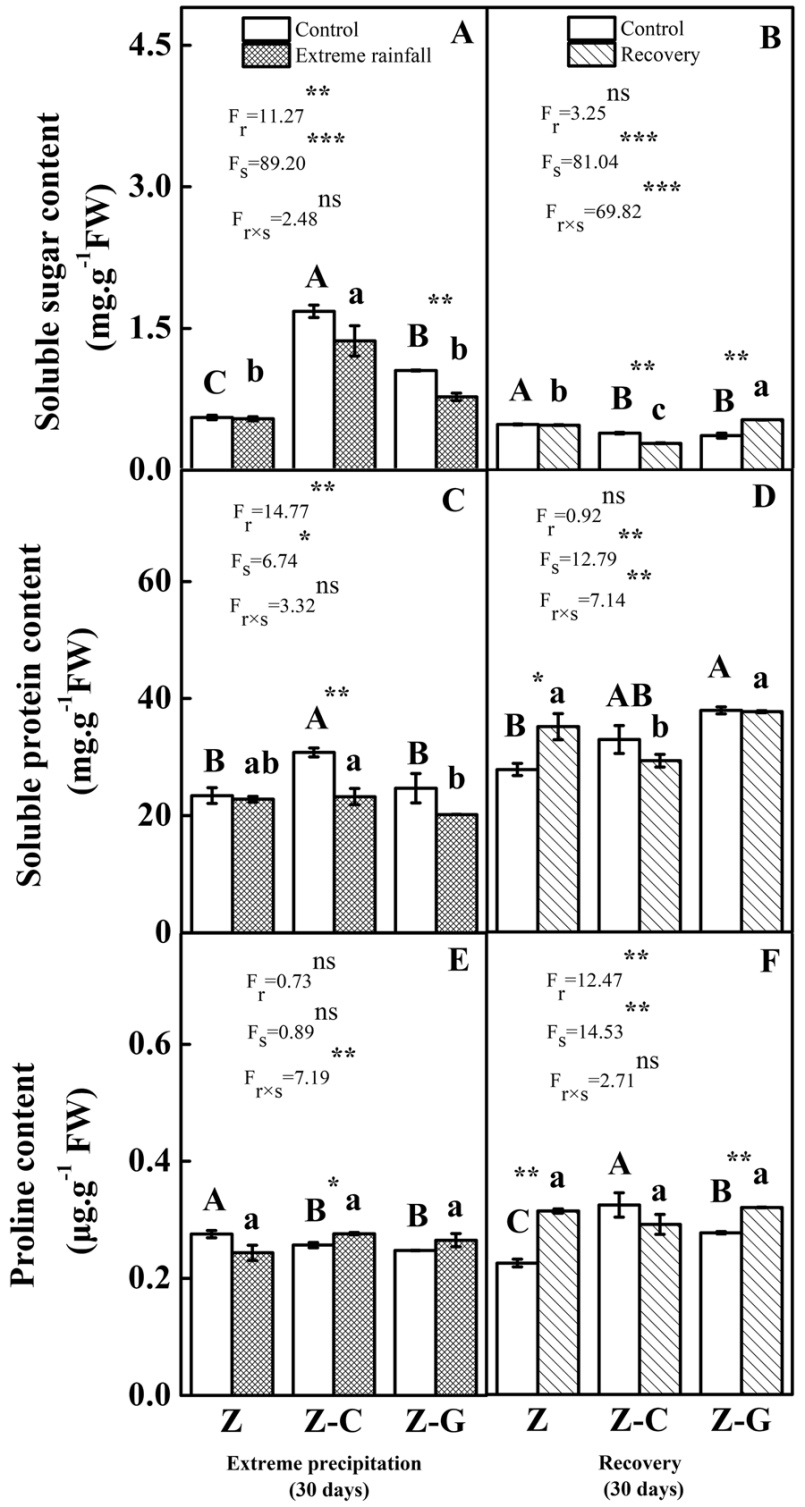
Effects of leaf osmotic adjustment substances on focal species after extreme rainfall and recovery. The left column represents 30 days of rainfall, the right column represents recovery for 30 days; “Z-G” denotes *Z*. *bungeanum* intercropping with *G*. *max*, “Z-C” denotes *Z*. *bungeanum* intercropping with *C*. *annuum*, and “Z” denotes the *Z*. *bungeanum* monoculture. Vertical bars show ± SE of the mean (n± SE). Different uppercase letters indicate significant differences between the control (normal rainfall) treatments; different lowercase letters indicate significant differences between the extreme rainfall treatments. “r” denotes extreme rainfall; “s” denotes planting system; “r × s” denotes the interaction of extreme rainfall and planting system; Proportion of explained variance by extreme rainfall and planting system effects and by their interactions (two-way ANOVA). Significant levels: *P < 0.05, **P < 0.01, ***P < 0.001, “ns” no significant.

Under normal rainfall, compared to the Z monoculture, the soluble sugar content of the *Z*. *bungeanum* leaves increased significantly and the proline content decreased significantly in the Z-C and Z-G mixed cultures (*P* < 0.05) (Fig. 3A,E). Compared with the Z and Z-G cultures, the soluble protein content of the *Z*. *bungeanum* leaves increased significantly in the Z-C culture (Fig. 3C). The extreme rainfall significantly decreased the soluble sugar content of *Z*. *bungeanum* in the Z-G mixed cultures. In the extreme rainfall treatment, the proline content of the *Z*. *bungeanum* leaves of the Z-C culture increased significantly (*P* < 0.05), while the soluble protein content of decreased significantly, compared to the control (*P* < 0.01). The interaction effects of planting systems and extreme rainfall significantly affected the proline content of the *Z*. *bungeanum* leaves (*P* < 0.01) (Fig. 3E).

After recovery with the previously normal rainfall treatment, the soluble sugar content of the *Z*. *bungeanum* leaves decreased significantly in the Z-C and Z-G mixed cultures (*P* < 0.05), and the soluble protein content increased significantly increased in the Z-G mixed culture, compared to the Z monoculture. The proline content increased significantly in the Z-C and Z-G mixed cultures than in the Z monoculture (*P* < 0.05). After recovery, compared to the control, the previously extreme rainfall treatment significantly increased the soluble sugar and proline content of the *Z*. *bungeanum* leaves in the Z-G mixed culture (*P* < 0.01) (Fig. 3B,F). The planting system significantly affected the soluble sugar, soluble protein and proline content of the *Z*. *bungeanum* leaves (Fig. 3B,D,F).

## Discussion

Extreme rainfall has been increasing globally and seriously affecting plant growth and yield^39^. The present study indicated that irrespective of the planting system, the LRWC and height of *Z*. *bungeanum* decreased under extreme rainfall compared to the control (Table 3). This was due to excessive water causing anoxia in the rhizosphere and hindering nutrient uptake by plants^40^. Many previous studies have shown that the presence of a legume has a positive effect on the growth of neighboring species^41–43^. Our results indicated that extreme rainfall did not significantly decreased the LRWC and height of *Z*. *bungeanum* in a legume mixed culture (*G*. *max*.), and it reduced the height of *Z*. *bungeanum* in the nonleguminous mixed culture (*C*. *annuum*) (Table 3). This could be attributed to the higher soil NH_4_^+^-N and NO_3_^−^-N in the legume mixed culture (as shown in our previous experiment^17^). In the Z-G mixed culture, *G*. *max* could maintain adequate physiological functioning in wet soils. Its moisture resistance property is associated with the formation of aeration tissue^44–46^. Our study found that the root biomass of *G*. *max* increased significantly under extreme rainfall (*P* < 0.05) (Table 3). The increase in root biomass provides the basis for the aeration of tissue and the nitrogen-fixing activity of leguminous crop^17,47^. Moreover, under extreme rainfall, the NH_4_^+^-N of *Z*. *bungeanum* leaves was higher in the Z-G mixed culture than in the Z monoculture and Z-C mixed cultures (*P* < 0.05) (Table 2). This indicates that under extreme rainfall, legumes facilitate focal species nutrient uptake via increasing N_2_ fixation^48^. After recovery, the LRWC of the focal species in three mixed cultures decreased compared to before the recovery. This may be due to the large diurnal temperature differences of the experiment site between different periods. Additionally, *Z*. *bungeanum* passes into a different growth phase, causing the lower LRWC.

Altered precipitation regimes influence plant eco-physiology by enhancing plant photosynthesis, transpiration and the leaf respiration rate^49,50^. Plant photosynthesis is a fundamental biological process and is greatly dependent on precipitation^51^. Previous studies have shown that increased precipitation could enhance plant photosynthesis and ecosystem carbon uptake^52^. An increase in precipitation can also indirectly affect focal species via its influence on the surrounding community^53^. The present study found that extreme rainfall significantly decreased the *P*_*n*_ of the focal species in three mixed cultures. Previous studies have revealed that the changes in *P*_*n*_ are mainly induced by the stimulation of stomatal conductance under increased precipitation^54^. Our results show that extreme rainfall significantly decreased *G*_*s*_ and *T*_*r*_, thereby resulting in the decreased *P*_*n*_ of focal species in the Z-C mixed culture. However, the *T*_*r*_ of focal species increased significantly in the Z-G mixed culture, which might be due to the formation of root aeration tissue of *G*. *max*^44,45,55,56^. After one month of rain recovery treatments, the *P*_n_ of focal species decreased compared with before recovery. This result may be due to the growth stage changes in *Z*. *bungeanum*. Moreover, when compared to August, the photosynthetic radiation in September declined. The *P*_n_ of the focal species in Z-C was lower compared with its control (*P* < 0.05). This clearly indicated that the photosynthetic resilience of focal species in Z-C mixed culture was not strong enough. This shows that legumes can alleviate the decline of photosynthetic function in *Z*. *bungeanum*.

The content of chlorophyll pigments in leaves is an indicator of plant physiological status^57^. Due to degradation of the chloroplast pigment in the Z monoculture and Z-C mixed cultures, extreme rainfall caused significant damage to the focal species. It has been previously demonstrated that, in the absence of any disturbance, pigment value also declines^58^. Nitrogen is critical for the growth and development of crop plants. The literature data shows that, in mixed stands, depending on the legume species and cultivar, from forage legumes to companion grasses, the rates of nitrogen transfer range from 0 to 73%^59^. Nitrogen fertilizer can increase chlorophyll and carotenoid content in the leaves of plants^60^; Sun *et al*.^17^ found that the soil nitrogen content in the Z-G mixed culture was significantly higher than in the Z monoculture. They also found that the leaf NH_4_^+^-N and NO_3_^−^-N content in the focal species of the Z-G mixed culture was significantly higher than that of the Z monoculture and Z-C mixed culture. Moreover, the leaf NH_4_^+^-N and NO_3_^−^-N content of *G*. *max* significantly decreased^17^. This proved that *G*. *max* could promote the nitrogen uptake of focal species. The present study shows that the Chl a, Chl b and Car content of the focal species in the Z-G mixed culture increased significantly under extreme rainfall. After recovery, the pigment content of the leaves of the focal species changed significantly in the Z monoculture and the Z-C mixed culture. This may be because, compared to the Z-G mixed culture, the pigment content of *Z*. *bungeanum* in the Z monoculture and Z-C mixed culture did not reach a steady state. This further proved that there was a significantly different impact on *P*_*n*_ in *Z*. *bungeanum* in the Z-C mixed culture (Table 4). However, in the Z-G mixed culture, there was no significant difference in the Chl a, Chl b, Car and Chl a/Chl b content of the focal species between the normal and extreme rainfall recovery treatments. These results indicate that the intercropping of leguminous crops could stabilize the pigmentation resilience of focal species.

Extreme rainfall causes a reduction in oxygen supply in the soil that further leads the over-production of ROS in plants^5,61^. The dramatic increase in the ROS level triggers protein degradation, lipid peroxidation, and deoxyribonucleic acid (DNA) fragmentation and causes cell death^62^. Our study found that, under extreme rainfall, when compared with their controls, the rates of O_2_^−·^. production were significantly higher in all cultures. The H_2_O_2_ content was significantly higher only in the Z monoculture, and no significant differences were observed in the Z-C and Z-G mixed cultures. Therefore, under extreme rainfall, the MDA content of the focal species increased significantly in the Z monoculture, while there was no significant difference in the MDA content of the focal species in the Z-C and Z-G mixed cultures. This demonstrated that increasing plant diversity could reduce the extreme rainfall damage to focal species.

Plants have developed antioxidant enzymes such as SOD and CAT required for the destruction of O_2_^−·^. and H_2_O_2_^63,64^. The SOD activity of *Z*. *bungeanum* was significantly enhanced in the Z-G mixed culture compared with its control. Moreover, the CAT activity of *Z*. *bungeanum* was the highest in the Z-G mixed culture in comparison with the Z-C mixed and Z monoculture cultures. This proved that species specificity plays a very important role in enhancing antioxidant enzyme activities to improve tolerance of focal species. The present findings are in line with a previous study that showed that facilitation should be more common when plants are subject to high abiotic conditions^65^. After recovery, the MDA content of focal species recovered to control levels for all cultures. Moreover, in extreme rainfall recovery treatments, the CAT activity of *Z*. *bungeanum* was significantly higher in Z-G compared with its control. That means that the damage from extreme rainfall on *Z*. *bungeanum* in the Z monoculture, Z-C and Z-G mixed cultures could be recovered from in 30 days. The present finding gives an indication of the strong tolerance to extreme rainfall of focal species in a legume mixed culture.

Osmoregulation is an important adaptation strategy to external stress^66^. Different patterns of carbohydrate accumulation in the leaves of flooded and control plants suggest that the decrease in water soluble carbohydrates in control plants is caused by the translocation from leaves to other plant organs^67^. Our results show that, under extreme rainfall, the soluble sugar content of focal species in the Z-G mixed culture was higher than that in the Z monoculture and Z-C mixed culture. This may suggest either a higher rate of soluble sugar or a high molecular weight fructan^68^. In addition, proline has several roles in osmotic adjustment, the elimination of ROS, and the maintance of cell redox status under stress^69^. Our study found that, under extreme rainfall the proline content of the focal species increased in the Z-C mixed cultures compared with their controls. This showed that proline accumulation in focal species might have a scavenging function^70^ and act as an osmolytes^71^. In recovery treatments, the soluble sugar and proline contents of focal species leaves in the Z monoculture and Z-G mixed culture were significantly increased compared with the control. The accumulation of these osmolytes represents an important adaptive response during the recovery period^72^.

## Materials and methods

### Study site

The experimental site was located in Mao county eastern Qinghai-Tibet Plateau (31°41′N, 103°53′E, elevation 1686 m). According to meteorological monitoring data from the Mao County Ecological Station of Chinese Academy of Science, the mean annual precipitation in the area is 920 mm, mean annual temperature is 8.9 °C and extreme minimum and maximum temperatures recorded are −11.6 °C, and 32.2 °C, respectively. The total precipitation in August is approximately 90 mm, and it is considered as the month with the most rainfall. According to a previous study, extreme rainfall is expected to increase in this area^73^. The soils are classified as Udic Luvisols^74^.

### Experimental design

A batch of uniform, two-year-old seedlings of *Z*. *bungeanum* were planted in April 2013. Six experimental treatments were set up as a randomized design with three replicates, with 18 plots of 2.6 m × 2.6 m spaced at least 1 m apart from each other. The three planting systems were as follows: (1) *Z*. *bungeanum* + *G*. *max* (Z-G); (2) *Z*. *bungeanum* + *C*. *annuum* (Z-C); (3) *Z*. *bungeanum* monoculture (Z). *G*. *max* and *C*. *annuum* were planted in April 2015. One *Z*. *bungeanum* was grown in the center of each plot, while species of *G*. *max* and *C*. *annuum* were planted at the same density (0.27 m^2^/individual) in all plots. No additional fertilization was applied after the experiment commenced, and the weeds in each plot were completely removed by hand each week. *Z*. *bungeanum* and intercrops were grown under natural rainfall before simulating extreme precipitation. In August 2015, we exposed our plots, to the precipitation treatment at random, in which triplicate plots per system received either normal (control) or extreme rainfall. To avoid external rainfall effects, all plots were kept under rainout shelters during the experimental period (from 1^st^ August to 30^th^ September 2015) to control soil moisture. To minimize greenhouse effects, the rainout shelters for each plot were situated 2 m aboveground^75^. Tap water was used to mimic extreme rainfall events, and a watering pot was used to compensate for rain.

Rainfall regimes were designated^76^, based on the average rainfall in the area during August of 3 mm/day (based on the average rainfall data during 1983–2013 from the Mao County Ecological Station of Chinese Academy of Science). This was designed as the control rain regime, while extreme rainfall was designated according to the abnormally high rainfall in August of 9.5 mm/day. Each planting system was first divided into two groups of different treatments: (1) Extreme rainfall (9.5 mm/day) and (2) Mean rainfall (Control, 3.0 mm/day). During the two-month-long experimental period, all the plots were watered in the morning (7–9 am) and evening (6–8 pm). After one month of extreme rainfall and control treatments, the systems were subsequently subjected to one month of recovery with rainfall of 3.0 mm/day. Around all plots, thick PVC panels were inserted to a depth of 0.5 m into the soil to prevent the lateral water movement between the plots and prevent interactions with roots from neighboring plots.

### Plant leaf collection

At the end of each stage, the youngest fully expanded *Z*. *bungeanum*, *G*. *max*, *and C*. *annum* leaves at the same developmental stage among plots were collected and placed in a liquid nitrogen container. The samples were taken back to the laboratory and stored at −80 °C to determine their physiological and biochemical parameters.

### Soil properties analysis

Soil sampling was taken randomly at 50 cm distance from the *Z*. *bungeanum* (in 0–10, 10–20, and 20–30 cm depth) in each plot^77^. The estimation of soil moisture content was performed gravimetrically by oven drying (105 °C for 24 h) 20 g of the soil sample. Soil NH_4_^+^-N and NO_3_^−^-N were determined with the help of a flow injection auto analyzer (AA3, Bran + Luebbe, Germany).

### Plant leaves nitrogen analysis

Approximately 0.1 g fresh leaves was ground and extracted in 1 mL of distilled water for 2.5 h. The NO_3_^−^-N content of the solution was determined with 10% (w/v) salicylic acid in 96% sulfuric acid. The values were quantified after generating a standard curve^78^. The NH_4_^+^-N content was determined with the colorimetric assay described by Krom^79^.

### Analysis of growth and biomass

Plant height (cm) was measured with the help of a measuring tape. The roots, shoots and leaves of neighboring plants were separated after digging the plants out from the soil, weighting, and subjecting them to oven drying at 70 °C for 24 h to measure dry weight. Determination of yield by weighing method.

### Determination of leaf relative water content

Leaf relative water content (LRWC)^80^ was determined and calculated according to the equation:

$${\rm{LRWC}}=[({\rm{FW}}\mbox{--}{\rm{DW}})/({\rm{TW}}\mbox{--}{\rm{DW}})]\times 100 \% $$

The expanded leaves were collected from each pot as samples and weighed to obtain their fresh weight (FW). Then, the samples were immediately dipped into deionized water, in dark conditions at room temperature. After 12 h, the leaves were weighted to obtain turgor weight (TW) and then subjected to oven drying at 70 °C for 24 h to determine the dry weight (DW).

### Pigment and Photosynthetic parameter

Chl a, Chl b, and Car were determined using 0.2 g of leaf sample and 5 mL of 100% acetone as a solvent. The samples were placed in dark conditions for 36 h at room temperature, and the extracting solution was determined spectrophotometrically at 662, 645, and 470 nm^81^. The value of Chl a/Chl b was calculated by dividing Chl a by Chl b. The *P*_n_, *G*_s_, *C*_i_ and *T*_*r*_ were measured for fully expanded leaves at similar development stages with a portable open-flow gas exchange system (LI-6400, LI-COR Inc., USA) during the late morning (9:00–11:00 h). The air relative humidity, CO_2_ concentration and photon flux density were maintained at 70–80%, 400 μmol mol^−1^ and 800 µmol m^−2^ s^−1^ respectively in all cases.

### Determination of biochemical parameters

The leaves (0.2 DW) were extracted three times with 6 ml of 80% ethanol at 80 °C for 30 min. The resulting supernatant was analyzed for soluble sugar by the modified anthrone method^82^. Proline was extracted with 2 mL of 10% acetic acid and 5 mL of 3% sulfosalicylic acid. The resulting supernatants were analyzed according to a previously described method^83^. Soluble proteins were determined using the Bradford G-250 reagent.

### Determination of ROS and lipid peroxidation

The production rate of O_2_^−·^. was measured by monitoring the nitrite formation from hydroxylamine in the presence of O_2_^−^ ^84^. Fresh leaves (0.2 g) were homogenized with 2 mL of 65 mM phosphate buffer (pH 7.8) and centrifuged at 5000 g for 10 min. The incubation mixture contained 0.9 mL of 65 Mm phosphate buffer (pH 7.8), 0.1 mL of 10 mM hydroxylammonium chloride and 1 mL of supernatant. After incubation at 25 °C for 20 min, 17 mM sulphanilamide and 7 mM α-naphthylamine were added to the incubation mixture, and kept at 25 °C for 20 min. The same volume of ethyl ether was added and centrifuged at 1500 g for 5 min. The absorbance wavelength of the aqueous solution was 530 nm. H_2_O_2_ was determined by monitoring the absorbance of the titanium-peroxide complex^85^. Fresh leaf tissue (0.2 g) was homogenized with 5 mL of acetone and centrifuged at 3000 g for 10 min. The reaction mixture contained 0.1 mL of titanium reagent (50 μL of 20% titanium tetrachloride in concentrated HCl), 0.2 mL of ammonia and 1 mL of supernatant, and was then centrifuged at 3000 g for 10 min. The resulting precipitate was washed five times with acetone and centrifuged at 10,000 g for 5 min. The precipitate was solubilized in 3 mL of 1 M sulfuric acid (H_2_SO_4_) and the absorbance was read at 410 nm. The MDA content was determined according to the thiobarbituric acid (TBA) method^86^. Fresh leaf tissue (0.2 g) was homogenized with 2 mL of 50 mM phosphate buffer (pH 7.8) and centrifuged at 12,000 g for 20 min. One milliliter of supernatant was mixed with 3 mL of 20% trichloroacetic acid (TCA) solution containing 2% TBA. The reaction mixture was incubated in a water bath at 95 °C for 30 min, rapidly cooled in an ice bath and then centrifuged at 15,000 g for 10 min. The absorbance was read at 532 and 600 nm. The amount of MDA was calculated using an extinction coefficient of 155 mM^−1^cm^−1^.

### Determination of antioxidant enzyme activities

The SOD activity was determined by the nitroblue tetrazolium (NBT) method^87^. One unit of SOD activity was defined as the quantity of enzyme required for 50% inhibition of NBT reduction at 560 nm. The CAT was determined using the methods of Fu^87^. For CAT, the decomposition of H_2_O_2_ was measured by the reduction in absorbance at 240 nm for 1 min. One unit of CAT activity was defined as an absorbance change of 0.01 units per min.

### Statistical analysis

Statistical analysis was performed using SPSS v.17.0 (SPSS Inc., Chicago, IL). One-way and two-way ANOVA were used to test the effects of planting systems and extreme rainfall on the soil properties and physio-biochemical parameters. Before the ANOVA, the data were checked for normality and the homogeneity of variances. Origin pro 8.5 was used for graphical presentation; error bars represent standard errors, and all data in the figures represent the mean ± SE.

## Conclusion

Extreme rainfall significantly impacted the growth and metabolism of *Z*. *bungeanum* in the three intercropping systems, especially for the Z monoculture. In *Z*. *bungeanum*, a significant increase in ROS (O_2_^−·^. and H_2_O_2_) and MDA content was found, while there were no significantly changes between the antioxidative (SOD and CAT) activities and the accumulation of osmoprotectants (proline, soluble sugars and soluble proteins) confirming its weak resistance potential in response to extreme rainfall. However, under extreme rainfall, intercropping with *G*. *max* had a significantly positive effect on the antioxidative activities, pigmentation and photosynthesis of *Z*. *bungeanum*. Intercropping with *C*. *annum* inhibited the growth, had a negative effect on *T*_*r*_, and decreased the CAT activity and soluble protein content of *Z*. *bungeanum*. After recovery, the soil NO_3_^−^-N significantly decreased in the Z-C mixed culture, resulting in a significant decrease in the *P*_*n*_ of *Z*. *bungeanum*. By increasing its root biomass, *G*. *max* enhanced soil NO_3_^−^-N in the Z-G mixed culture and improved the *T*_*r*_ and CAT activity of *Z*. *bungeanum*. Legumes could enhance the tolerance of *Z*. *bungeanum* to extreme rainfall. The present findings provide baseline information about the effects of neighbor species (leguminous and nonleguminous) on focal species under extreme rainfall stress. However, more detailed studies are required to explore the interactive mechanisms of different agroforestry plant species, which could lead to a better understanding of the possible physiological responses in coping with future climate change.
